# Supplementary material for: Drosophila Evi5 is a critical regulator of intracellular iron transport via transferrin and ferritin interactions
Source: Nat Commun. 2024 May 14;15:4045. doi: 10.1038/s41467-024-48165-9 (PMC11094094; doi:10.1038/s41467-024-48165-9)
Supplement: Supplementary file 1 — Supplementary Information [file 41467_2024_48165_MOESM1_ESM.pdf]

Supplementary Information

"*Drosophila* Evi5 is a Critical Regulator of Intracellular Iron Transport via Transferrin and Ferritin Interactions"

Soltani et al.

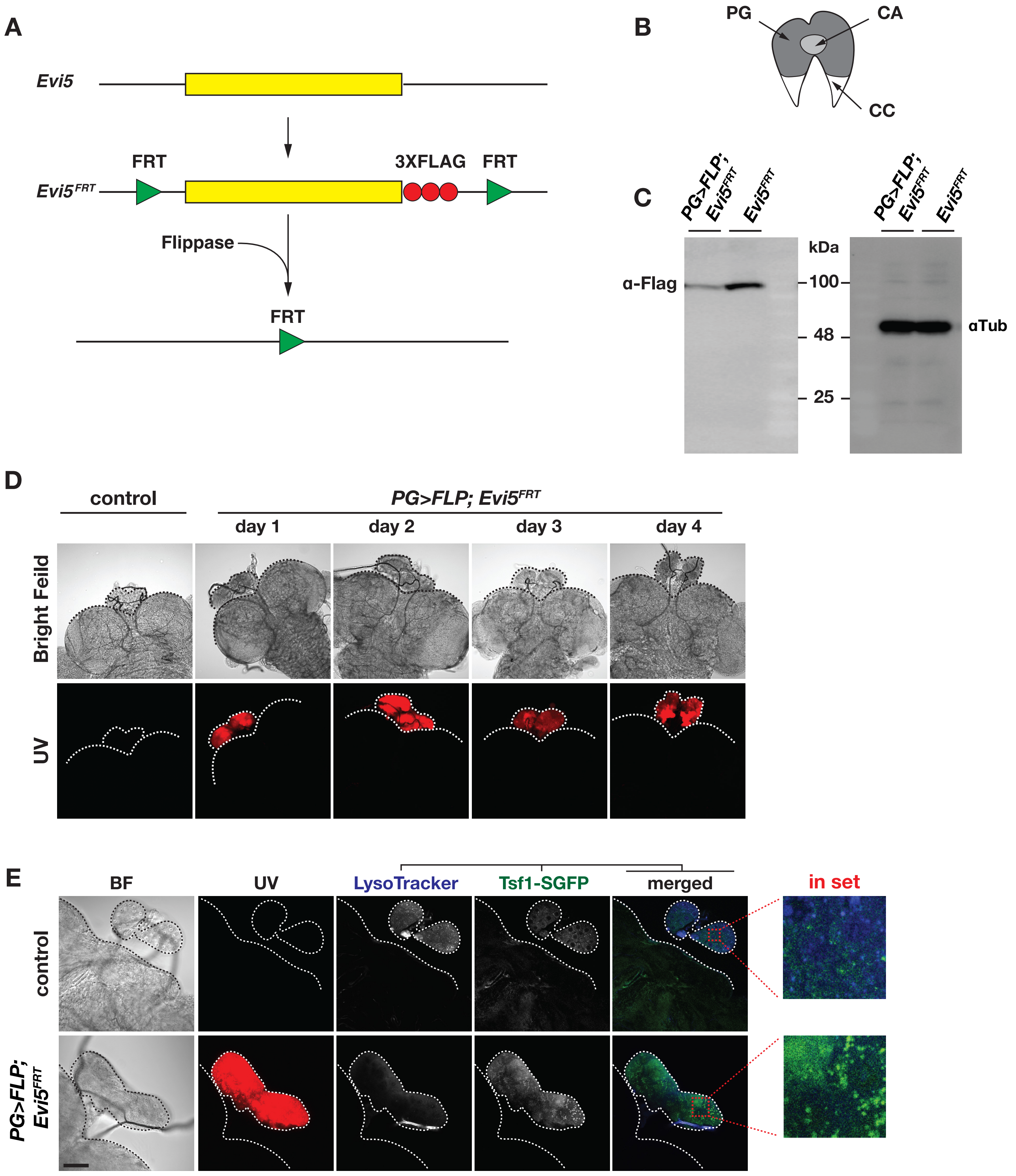

Soltani et al., Figure S1

Figure S1. Perdurance of red autofluorescence in cultured *Evi5^{FRT}* BRGCs.

A. Schematic representation of the *Evi5^{FRT}* knock-in allele. Endogenous Evi5 was replaced with an allele that is flanked with FRT (Flippase Recombinase target) sites (green triangles) and encodes 3x Flag epitope tags (red circles). The *Evi5* transcription unit can be conditionally excised by tissue-specific expression of *Flippase (FLP)*. **B.** Schematic illustration of the larval ring gland (RG). The RG is a tripartite tissue composed of the Prothoracic Gland (PG), Corpus Allatum (CA), and Corpus Cardiacum (CC). We achieved PG-specific excision of the *Evi5* transcription unit (see A) via a PG-specific Gal4 driver (abbreviated PG>) that induces the expression of Flippase in the PG, but not the CA and the CC. **C.** Western blot analysis of FLAG-Evi5 and alpha-Tubulin (αTub) in *Evi5^{FRT}* and *PG>FLP;Evi5^{FRT}* ring glands. Alpha-Tub is used as a loading control. **D.** Dissected BRGCs from *PG>FLP;Evi5^{FRT}* were kept in Schneider medium for four days and monitored for the presence of autofluorescence caused by protoporphyrin accumulation. Control ring gland isolated from *PG>w^{1118}* animals. **E.** Lysosome and Tsf1-sGFP co-localization in BRGCs. BRGCs of control and *PG>FLP;Evi5^{FRT}* larvae were co-cultured with fat bodies isolated from *Tsf1-sGFP* larvae. BRGCs and fat bodies were maintained in Schneider S2 cell medium for 48 hours and subsequently stained with LysoTracker Blue. The blue channel displays lysosomes, while the grey and green channels represent Tsf1. Detail images indicate selected regions (red-dotted box) in the merged channel. Scale bars = 150 μm. UV: ultraviolet light, BF: bright field.

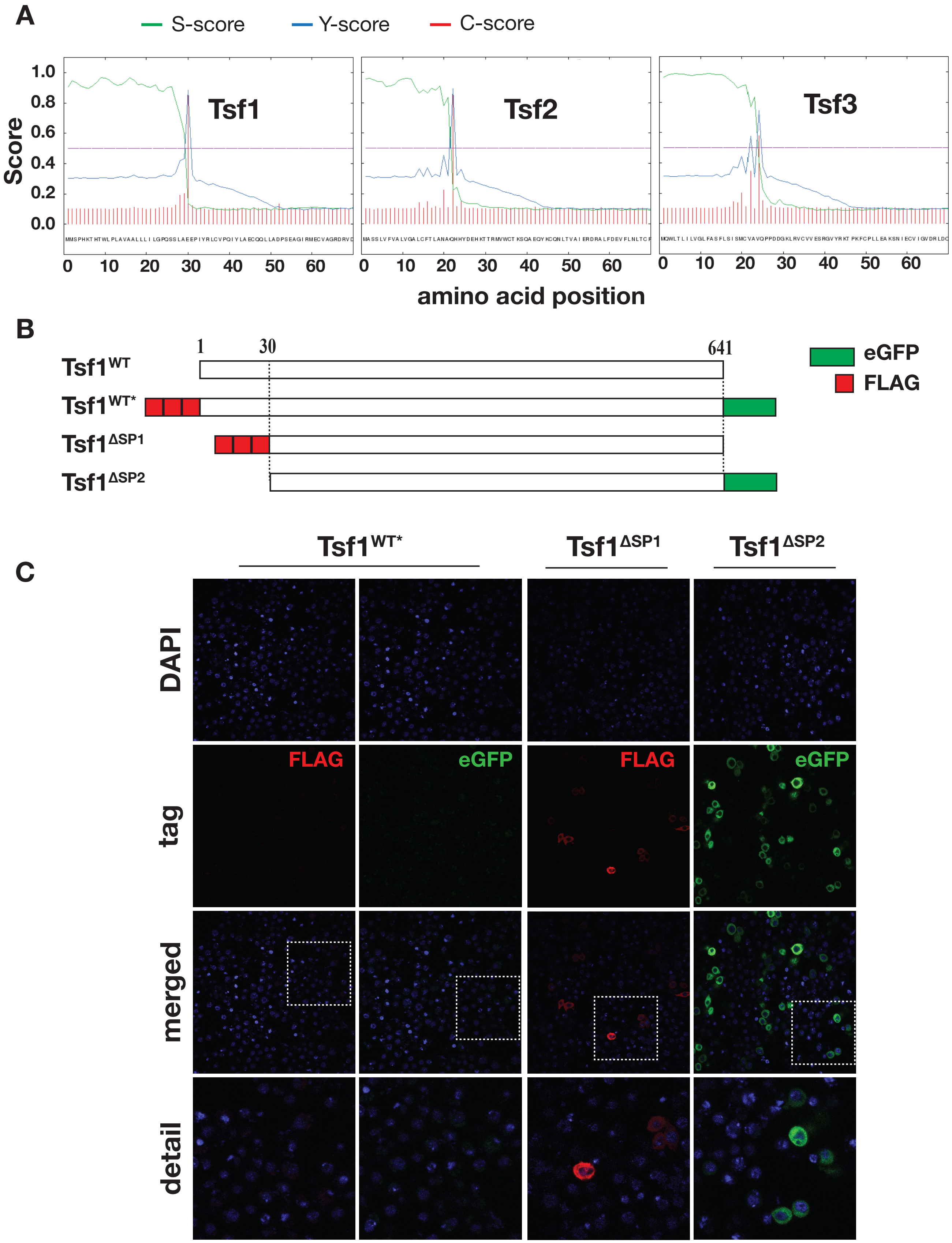

Soltani et al., Figure S2

Figure S2. Tsf proteins contain N-terminal secretion signal

A. Signal-P analysis of Tsf1, Tsf2 and Tsf3 proteins. The S-score indicates the secretion score (green), the Y-score (blue) shows the likelihood of a true positive cleavage site in a protein, and C-score represents the cut site score (red). The Y-axis shows the value of S, Y and C scores and the X-axis represents the amino acid position. **B.** S2 cell transfection plasmid design for Tsf1 protein variants. The $Tsf1^{WT*}$ plasmid harbours a wild-type Tsf1 cDNA plus a sequence encoding three N-terminal Flag epitopes (red boxes) and a C-terminal section encoding eGFP. $Tsf1^{\Delta SP1}$ and $Tsf1^{\Delta SP2}$ plasmids lack sequences encoding the secretion signal peptide (1- 30 amino acids) but are encoding distinct tags, resulting in $Tsf1^{\Delta SP1}$ harbouring N-terminal 3xFlag epitopes and $Tsf1^{\Delta SP2}$ being fused to C-terminal eGFP. **C.** S2 cells transfected with $Tsf1^{WT*}$ and $Tsf1^{\Delta SP1}$ and $Tsf1^{\Delta SP2}$ plasmids. The blue channel shows DAPI (DNA stain). Red and green channels show Flag and eGFP signals, respectively. The detail panels correspond to the white boxed areas in the in the “merged” panels.

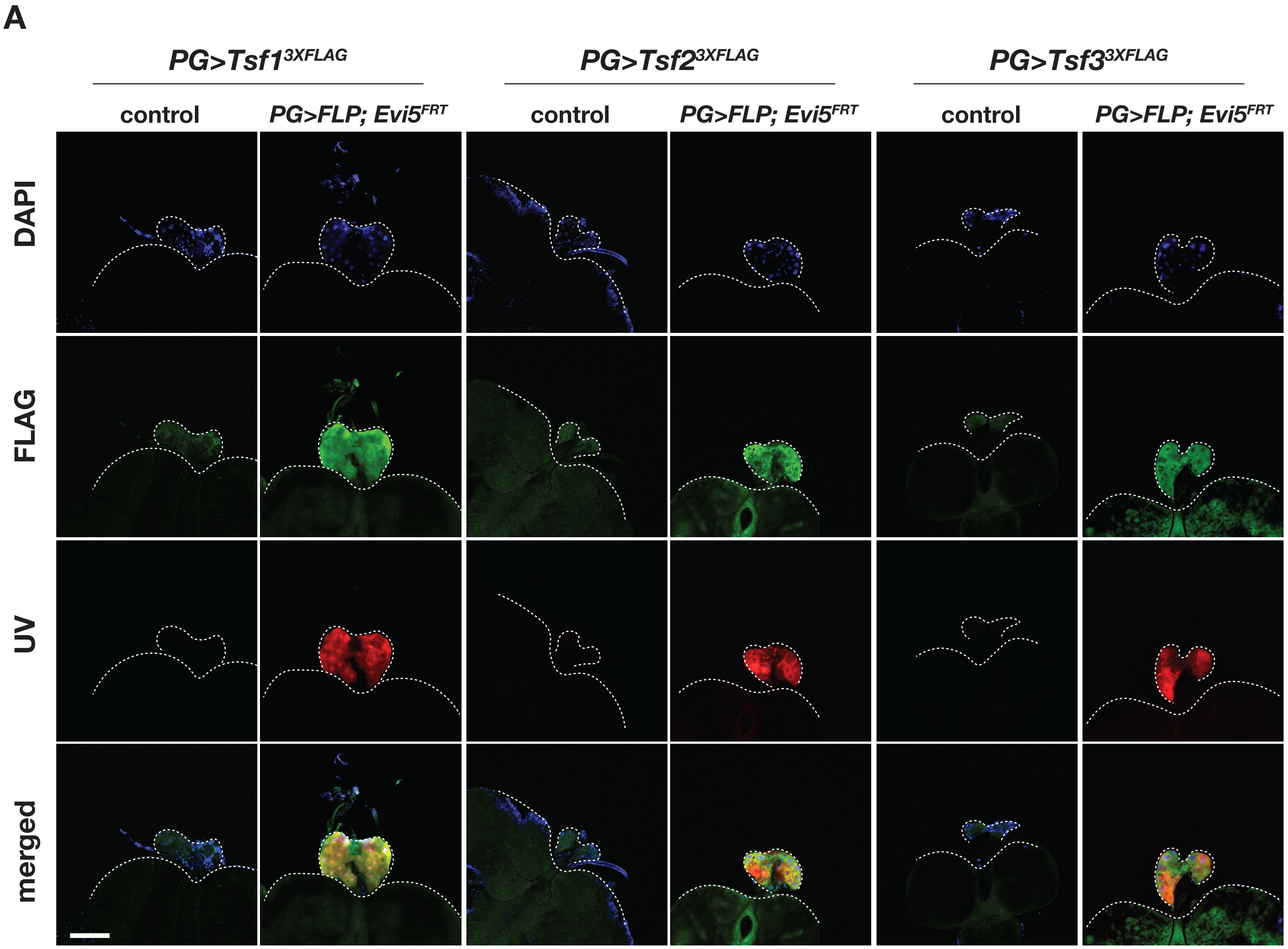

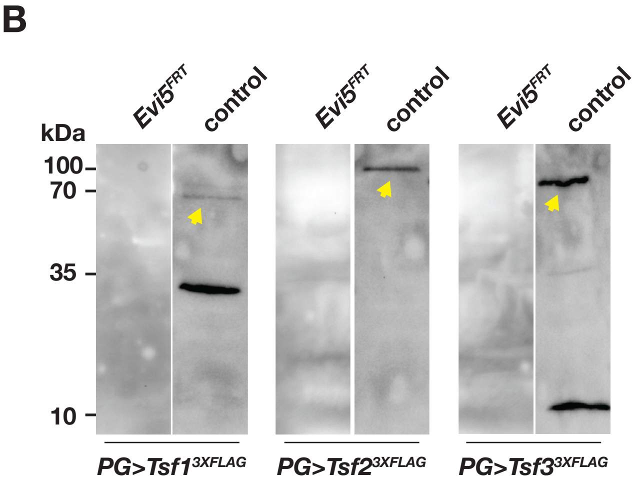

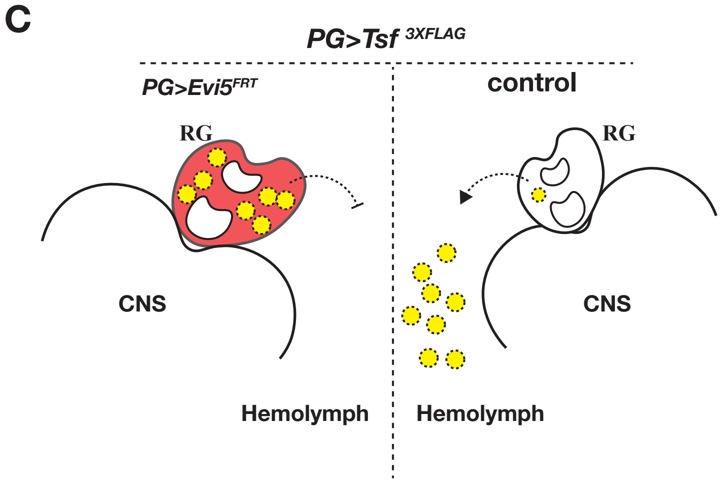

Soltani et al., Figure S3

Figure S3. PG-specific depletion of *Evi5* blocks secretion of Tsf proteins into hemolymph.

A. Immunolocalization of 3x-Flag-tagged Tsf1, Tsf2 and Tsf3 (green) in $Evi5^{FRT}$ mutant ($PG>FLP;Evi5^{FRT}$) and control backgrounds ($PG>Tsf1^{3XFLAG}$, $PG>Tsf2^{3XFLAG}$, $PG>Tsf3^{3XFLAG}$). The blue channel represents DAPI (DNA stain), and the red channel (UV) shows the presence of absence of autofluorescence in the prothoracic gland resulting from loss of Evi5 function and control backgrounds. Scale bar = 250 µm. **B.** Western blot analysis of hemolymph samples derived from Flag-tagged Tsf1, Tsf2 and Tsf3 in a $PG>FLP;Evi5^{FRT}$ and controls. Yellow arrows indicate secreted transferrin (Tsf) proteins from the PG into the hemolymph. **C.** A model for Evi5 function in Tsf protein secretion and trafficking in the PG. PG-specific loss of *Evi5* function blocks secretion of Tsf proteins into the hemolymph and causes autofluorescence due to heme precursor accumulation.

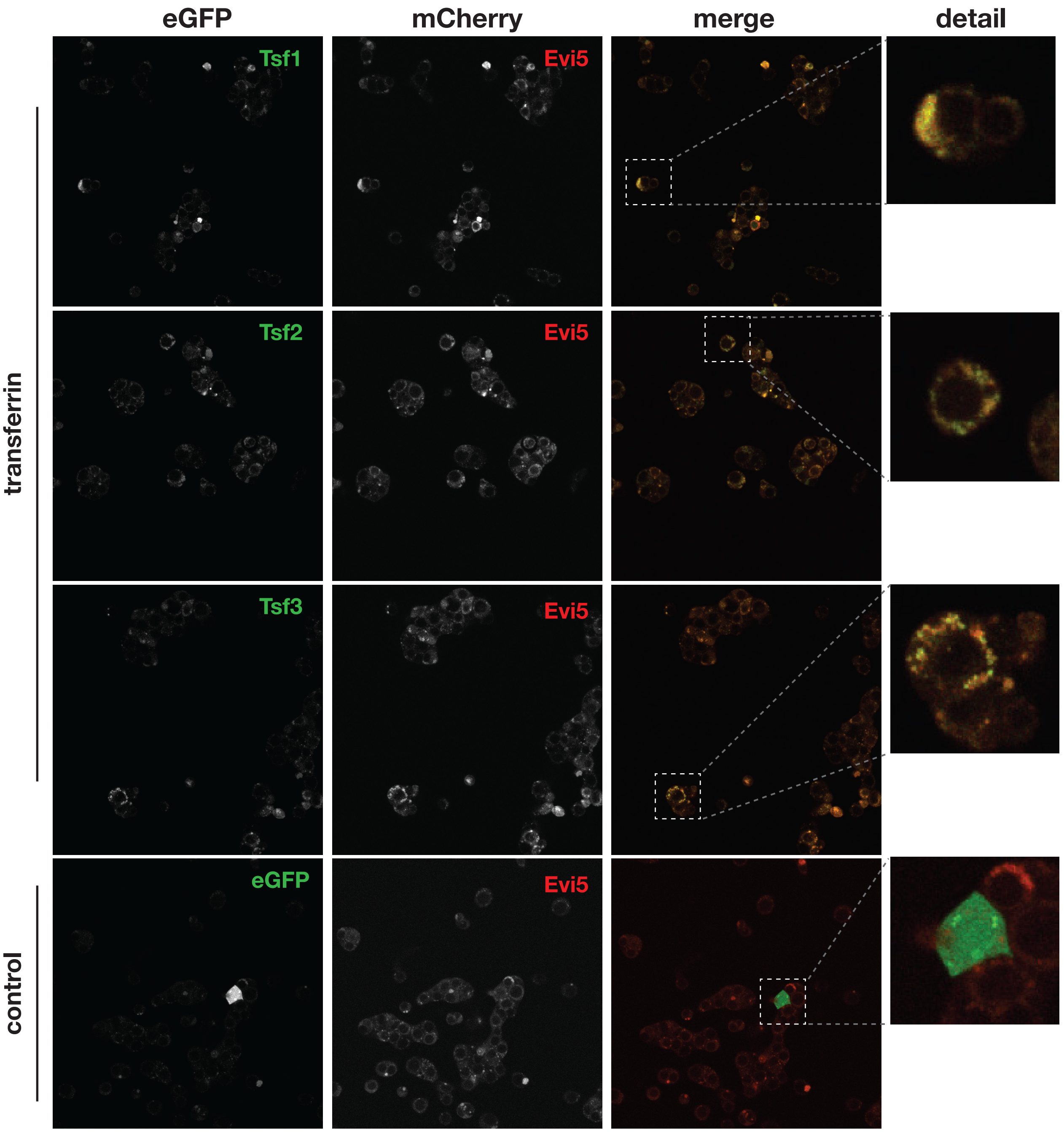

Soltani et al., Figure S4

Figure S4. Evi5 colocalizes with Tsf1, Tsf2, and Tsf3.

Images of S2 cells co-transfected with Evi5-mCherry and one of the following plasmids encoding i) Tsf1-eGFP, ii) Tsf2-eGFP, iii) Tsf3-eGFP or iv) eGFP. Evi5 is tagged with mCherry, but otherwise wild type. The secretion signals of Tsf1, Tsf2 and Tsf3 were removed, and all three proteins were tagged with eGFP. Images captured in the red and green channels are shown in grayscale, but all merged images are shown in color to visualize co-localized proteins in yellow. Areas in the white boxes are enlarged to the right ("detail").

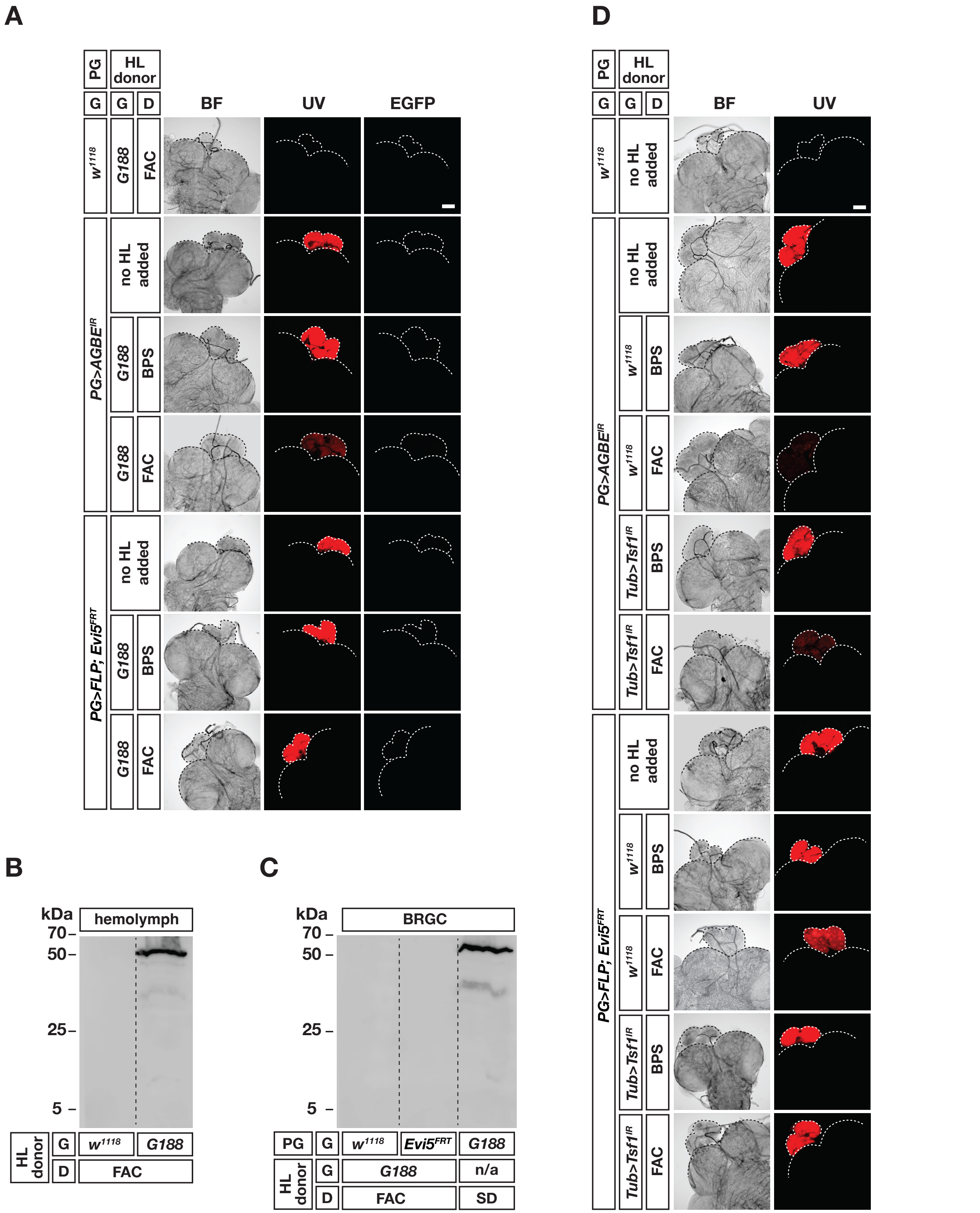

A
PG
HL donor
G
G
D
BF
UV
EGFP
w1118
G188
FAC
PG>AGBEIR
no HL added
G188
BPS
G188
FAC
PG>FLP; Evi5FRT
no HL added
G188
BPS
G188
FAC
B
kDa
hemolymph
70
50
25
5
HL donor
G
w1118
G188
D
FAC
C
kDa
BRGC
70
50
25
5
PG
G
w1118
Evi5FRT
G188
HL donor
G
G188
n/a
D
FAC
SD
D
PG
HL donor
G
G
D
BF
UV
w1118
no HL added
PG>AGBEIR
no HL added
w1118
BPS
w1118
FAC
Tub>Tsf1IR
BPS
Tub>Tsf1IR
FAC
PG>FLP; Evi5FRT
no HL added
w1118
BPS
w1118
FAC
Tub>Tsf1IR
BPS
Tub>Tsf1IR
FAC

Figure S5. Examining whether ferritin delivers iron directly to the PG.

A. Autofluorescence caused by protoporphyrin accumulation in prothoracic glands ("PG") of cultured brain ring gland complexes (BRGCs) incubated in the presence or absence of larval hemolymph ("HL") collected from $Fer1HCH^{G188}$ ("*G188*") larvae, which produce FerHCH-GFP fusion proteins. Genotypes ("G") of incubated prothoracic glands were w^{1118} (controls), $PG>AGBE^{IR}$ and $PG>FLP;Evi5^{FRT}$. Larvae used for obtaining hemolymph were reared on diets ("D") supplemented with either BPS (an iron chelator) or FAC (an iron source). Scale bar = 150 µm. BF = bright field image. UV: ultraviolet light. eGFP: enhanced Green Fluorescent Protein. **B.** Western blot analysis of hemolymph ("HL") samples from w^{1118} (control) and $Fer1HCH^{G188}$ ("*G188*") larvae raised on iron-enriched media ("FAC"). Anti-GFP antibodies were used to detect Fer1HCH-GFP fusion proteins. **C.** Western blot analysis of BRGCs isolated from w^{1118} (controls), $PG>FLP;Evi5^{FRT}$ ("$Evi5^{FRT}$") co-incubated with $Fer1HCH^{G188}$ ("*G188*") hemolymph ("HL") isolated from larvae. BRGC complexes dissected from $Fer1HCH^{G188}$ larvae served as a positive control. Anti-GFP antibodies were used to detect Fer1HCH-GFP fusion proteins. **D.** Autofluorescence caused by protoporphyrin accumulation in prothoracic glands ("PG") of cultured BRGCs incubated in the presence or absence of larval hemolymph ("HL") collected from w^{1118} or $\alpha Tub84B>Tsf1^{IR}$ larvae. Hemolymph donor larvae were reared on BPS- or FAC-supplemented diets and hemolymph was collected from 3rd instar larvae. Genotypes of incubated BRGCs were w^{1118} (controls), $PG>FLP;Evi5^{FRT}$ and $PG>AGBE^{IR}$. Scale bar = 150 µm.

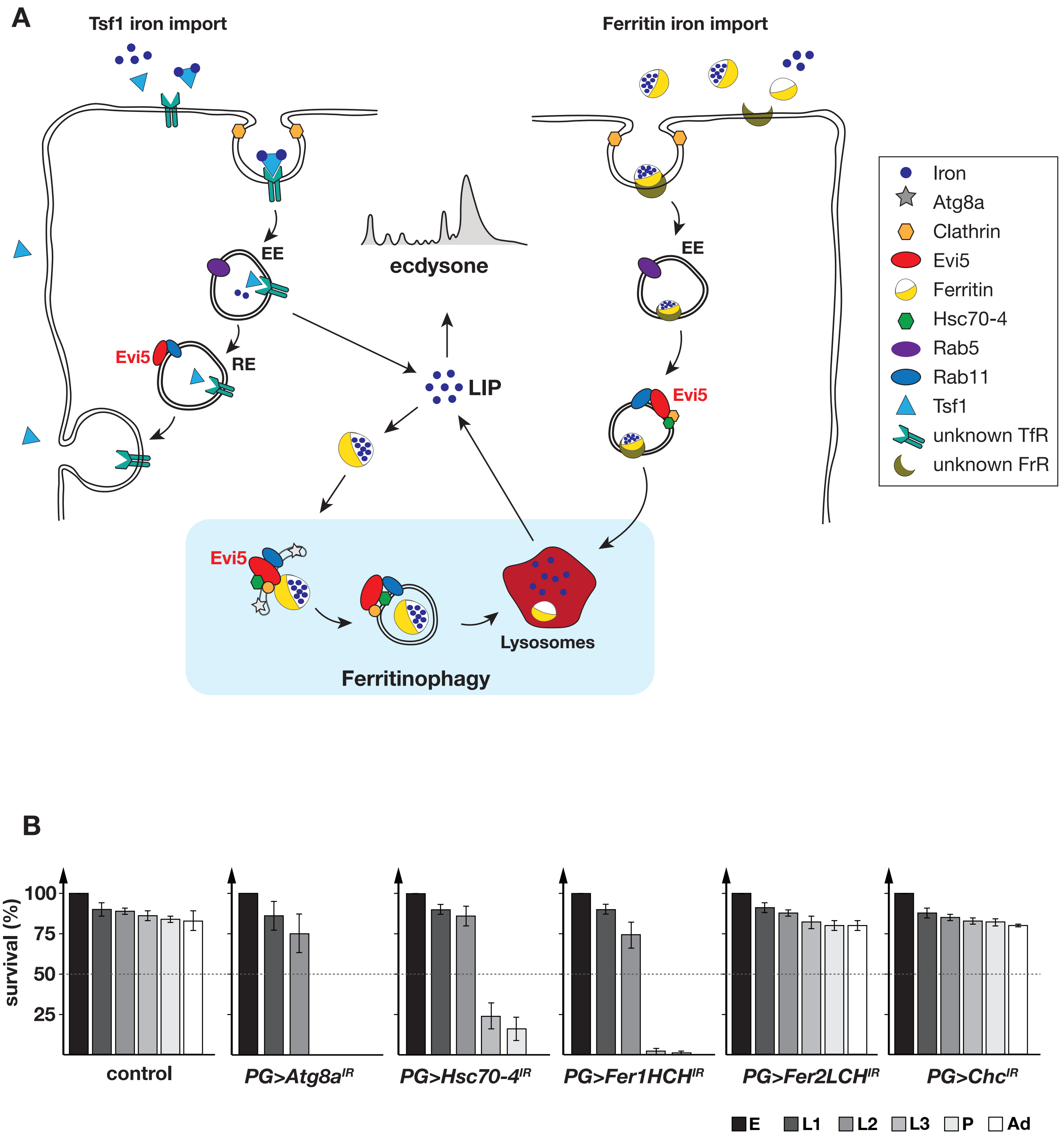

Soltani et al., Figure S6

Figure S6. Model for Evi5 function and RNAi survival study targeting ferritinophagy candidate genes

A. Model for Evi5 function. left: steady-state iron delivery pathway mediated by Transferrin 1 (Tsf1). While this pathway addresses the baseline iron requirement of cells, it may be insufficient to meet the peak iron demands during ecdysone synthesis. In the steady state, iron-loaded Transferrin 1 (holo-Tsf1) enters cells by binding to an unidentified receptor (TfR), with Rab5 controlling its entry into early endosomes (EE). In endosomes, iron is released through acidification and transported into the cytoplasm. Apo-Tsf1 and its receptor are then recycled to the plasma membrane via recycling endosomes (RE), regulated by Evi5 and Rab11. Labile iron pool (LIP) fuels heme and Fe-S cluster synthesis. Excess iron is stored in ferritin, which in *Drosophila* is mainly present in the hemolymph, serving as an extracellular iron source for high-demand tissues. right: The ferritin-mediated iron delivery pathway. Ferritin releases stored iron during high cellular iron demand through lysosomal degradation, known as ferritinophagy. This process can provide substantially more iron compared to Tsf1-mediated uptake. We propose that Evi5 and Rab11, along with unidentified autophagy proteins (Atg), facilitate this process. Iron released from lysosomes replenishes the LIP, supporting essential processes like heme and Fe-S cluster biosynthesis. During peak demand, holo-ferritin must enter cells through early endosomes (EE) using an unknown receptor, likely mediated by Rab5. Endosomal vesicles, regulated by Evi5 and Rab11, then target holo-ferritin to lysosomes. **B.** Survival study examining development stages: embryo (E), 1st instar (L1), 2nd instar (L2), 3rd instar larvae (L3), pupae (P) and adults (Ad). PG-specific RNA interfering with controls (*PG*>w^{1118}), Autophagy-related 8a (*Atg8a*), Heat shock protein 70 cognate 4 (*Hsc70-4*), Ferritin 1 heavy chain (*Fer1HCH*), Ferritin 2 light chain (*Fer2LCH*) and Clathrin heavy chain (*Chc*) function (ferritinophagy candidate genes). Error bars indicate standard error, with the center indicating the average. Three biological replicates tested (each = 50 individuals). The dotted line denotes 50% survival. Source data are provided (Source Data file).

corresponding to Figure 6

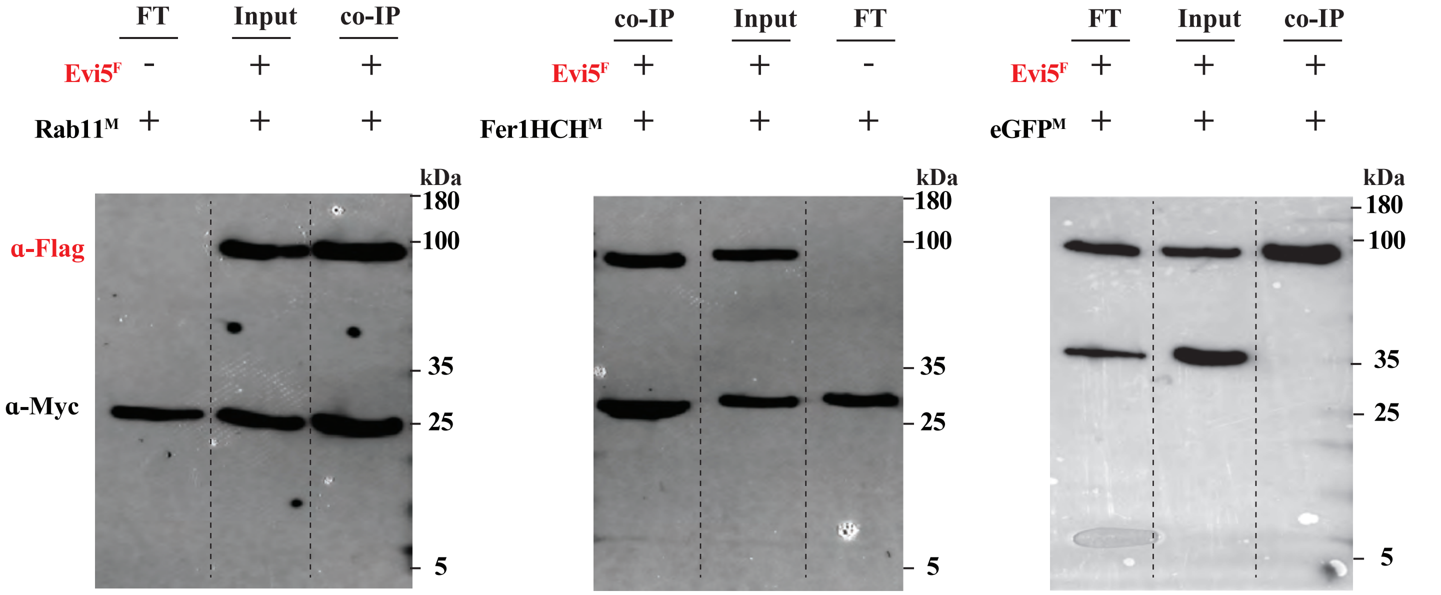

corresponding to Figure S3

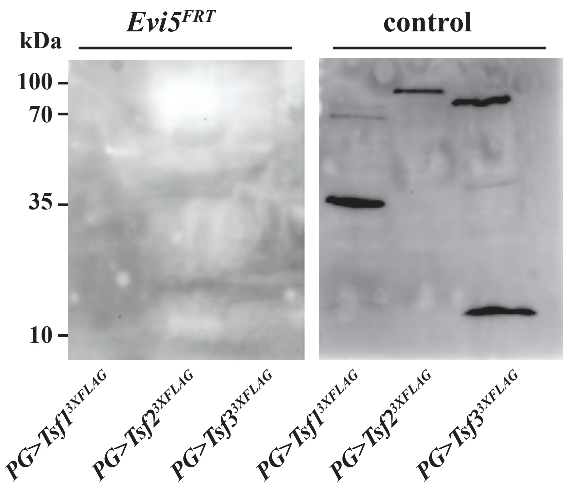

corresponding to Figure S5

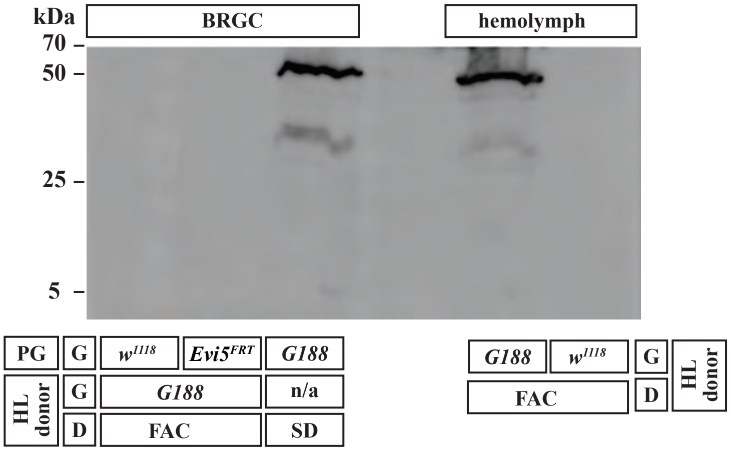

Figure S7. Uncropped Western blots corresponding to Figure 6, S3 and S6.

Uncropped Western blots corresponding to Fig. 6B, Fig. S3B and Fig. S5B and C.

Table S1. List of fly stocks used in this study.

Name^{A}	Stock ID^{A}	Name	Stock ID
UAS-Evi5^{IR1}	105146	*UAS-Bulli*^{IR}	61915
UAS-Evi5^{IR2}	38350	*UAS-ParCas*^{IR}	57301
UAS-Tsf1^{IR}	106479	*UAS-Sbf*^{IR}	62898
UAS-Rab1^{IR}	27299	*UAS-CG1695*^{IR}	57223
UAS-Rab2^{IR}	34922	*UAS-CG4041*^{IR}	110700
UAS-Rab3^{IR}	31691	*UAS-CG4552*^{IR}	32333
UAS-Rab4^{IR}	33757	*UAS-CG5337*^{IR}	77447
UAS-Rab5^{IR}	103945	*UAS-Wkd*^{IR}	32394
UAS-Rab6^{IR}	35744	*UAS-CG5745*^{IR}	110561
UAS-Rab7^{IR}	27051	*UAS-CG5916*^{IR}	110396
UAS-Rab8^{IR}	34373	*UAS-GAPsec*^{IR}	77400
UAS-Rab9^{IR}	42942	*UAS-CG6182*^{IR}	55328
UAS-Rab9D^{IR}	109376	*UAS-Rab3-GAP*^{IR}	34976
UAS-Rab9Db^{IR}	38269	*UAS-CG7112*^{IR}	32929
UAS-Rab9Fa^{IR}	109230	*UAS-CG7324*^{IR}	100125
UAS-Rab9Fb^{IR}	34374	*UAS-CG7742*^{IR}	28670
UAS-Rab10^{IR}	26289	*UAS-RN-tre*^{IR}	108444
UAS-Rab11^{IR}	108382	*UAS-CG8155*^{IR}	24101
UAS-Rab11^{CA}	9791	*UAS-CG8449*^{IR}	108736
UAS-Rab14^{IR}	28708	*UAS-Sky*^{IR}	34859
UAS-Rab18^{IR}	27665	*UAS-CG11490*^{IR}	33729
UAS-Rab19^{IR}	34607	*UAS-CG12241*^{IR}	107134
UAS-Rab21^{IR}	29403	*UAS-CG16896*^{IR}	28776
UAS-Rab23^{IR}	28025	*UAS-Tbc*^{IR}	53011
UAS-Rab26^{IR}	31177	*UAS-Muc14A*^{IR}	66313
UAS-Rab27^{IR}	50537	*UAS-Plx*^{IR}	62385
UAS-Rab30^{IR}	31120	*UAS-CG42795*^{IR}	32848
UAS-Rab32^{IR}	28002	*UAS-Hsc70-4*^{IR}	101734
UAS-Rab35^{IR}	28342	*UAS-Fer1HCH*^{IR}	102406
UAS-Rab39^{IR}	53995	*UAS-Fer2LCH*^{IR}	106960
UAS-Rab40^{IR}	29579	*UAS-Atg8a*^{IR}	109654
UAS-RabX1^{IR}	28033	*UAS-Chc*^{IR}	103383
UAS-RabX2^{IR}	32360	*UAS-Ppox*^{IR}	100577
UAS-RabX4^{IR}	28704	*UAS-Flp*	4539
UAS-RabX5^{IR}	28045	*UAS-Evi5*^{WT}*-mVenus*	-
UAS-RabeX-5^{IR}	50573	*UAS-Evi5*^{R160A}*-mVenus*	-
UAS-RabX6^{IR}	26281	*UAS-Tsf1-3xFLAG*	-
UAS-Msp-300^{IR}	103378	*UAS-Tsf2-3xFLAG*	-
UAS-Bulli^{IR}	63531	*UAS-Tsf3-3xFLAG*	-
UAS-Mon1^{IR}	103378	*Evi5*^{FRT}	-
UAS-Sprint^{IR}	80367	*phm22-GAL4*	-
UAS-CCZ1^{IR}	62889	*aTub84B-GAL4*	5138
UAS- Crag^{IR}	53261	*Tsf1-SGFP*	318585

^{A} **IR:** “Inverted Repeat”, indicates RNAi line, **CA**: constitutively active form. Stock IDs are from Bloomington *Drosophila* Stock Center and Vienna *Drosophila* Resource Center.

Table S2. Primers used in this study.

Name	Sequence (5'-3')
**qPCR primers**	
RP49-Fwd	CGGATCGATATGCTAAGCTGT
RP49-Rev	GCGCTTGTTCGATCCGTA
ALAS-Fwd	ACCAACGGAACGTCTCCTAC
ALAS-Rev	CTTCGACGGGGAAACCTT
Neverland-Fwd	CCCTCACCTAGGAGCCAACT
Neverland-Rev	GGCATATAACACAGTCGTCAGC
Shroud_Fwd	CGAATCGCTGCACATGAC
Shroud-Rev	TAGGCCCTGCAGCAGTTTAG
Spookier-Fwd	GCGGTGATCGAAACAACTC
Spookier-Rev	CGAGCTAAATTTCTCCGCTTT
Cyp6t3-Fwd	GGTGTGTTTGGAGGCACTG
Cyp6t3-Rev	GGTGCACTCTCTGTTGACGA
Phantom-Fwd	GGCATCATGGGTGGATTT
Phantom-Rev	CAAGGCCTTTAGCCAATCG
Disembodied-Fwd	GTGACCAAGGAGTTCATTAGATTTC
Disembodied-Rev	CCAAAGGTAAGCAAACAGGTTAAT
Shadow-Fwd	CAAGCGGATATTTGTAGACTTGG
Shadow-Rev	AAGCCCACTGACTGCTGAAT
Shade-Fwd	CCGCATTCAGCAGTCAGTGG
Shade-Rev	ACCTGCCGTGTACAAGGAGAG
Rab5-Fwd	TTGTGAAGATTTGAAAACGACT
Rab5-Rev	TGCTGAGTAAGTCTTTCTGCTAAGA
Rab11-Fwd	ACGCGCAGCATAGAGGTC
Rab11-Rev	CGGTAGTAGGCAGAGGTGATG
Fer1HCH-Fwd	TGCTAGCCTGCTCCTGTTG
Fer1HCH-Rev	GTCCACCCAGTCCTTGGTAA
Fer2LCH-Fwd	CGCTCTCCAAAACCACACA
Fer2LCH-Rev	GTGAAACTCTGAAAATCAACTGCT
**Evi5^{FRT}**	
gRNA 1-Fwd	ATCCGGGTGAACTTCGTTGAAATATCTATCACATGTGTTTTAGAGCTAGAAATAGCAAG
gRNA 1-Rev	TTTCTAGCTCTAAAACCAACCATAAGAAATCAAATACGACGTTAAATTGAAAATAGGTC
gRNA 2-Fwd	ATCCGGGTGAACTTCGTACAAAACCCGTGCTTATAAGTTTTAGAGCTAGAAATAGCAAG
gRNA 2-Rev	TTTCTAGCTCTAAAACACCATCCCATCCTCCATAGTCGACGTTAAATTGAAAATAGGTC
Evi5-genomic locus1-Fwd	gaagcaggtggaattcCCGCCTAGGCAATCAACAG
Evi5-genomic locus1-Rev	cgataagcttggatccTTTCTAGAGAATAGGAACTTCACATG
Evi5-genomic locus2-Fwd	gttcctattctctagaaaGGTGACTAGCTGACAAGC
Evi5-genomic locus2-Rev	cgataagcttggatccAAATGACCGAGACATGGG
Evi5-genomic locus3-Fwd	tggacgagctgtacaagtaaATTCTCTAGAAAGTATAGGAACTTC
Evi5-genomic locus3-Rev	atctagagtcgcggccccctaCTCCTTGAGCTTCTTGTG
Add FRT site-Fwd	gcgatcgcGAAGTTCCTATTCTCTAGAAAgtataggaacttcAGAAAGGTACCGGTTCCG
Add FRT site-Rev	agaataggaacttcgtcgaCTCCGGAttaattaaACATGATTAACCCTCACTAAAGG
Add FRT site-Fwd	tcgacGAAGTTCCTATTCTCTAGAAAgtataggaacttcCCCTTTAGTGAGGGTTAATTC
Add FRT site-Rev	aataggaacttcgcgatcgcAGAAGACcatatgGTATCCGCTCATGAGATTATC
Backbone 1-Fwd	TTACTTGTACAGCTCGTCCATG
Backbone 1-Rev	TAGGGGCCGCGACTCTAG
Backbone 2-Fwd	TTTCTAGAGAATAGGAACTTCAC
Backbone 2-Rev	GGATCCAAGCTTATCGATTTC
Backbone 3-Fwd	GAATTCCACCTGCTTCAG
Backbone 3-Rev	GGATCCAAGCTTATCGATTTC
Sequencing 1	GCGACACGGAAATGTTGAATAC
Sequencing 2	AAACCCGCCTAAGAATGTG
Sequencing 3	CGTGGTAAAGTTGAACATACC
Sequencing 4	TGGAATACGTTCTAGTTCCGATC
Sequencing 5	GCCTTGTAATCGTATTTTACACG
Sequencing 6	GCGACACGGAAATGTTGAATAC
Sequencing 7	TAAGCTCATCGAGAGCGATG
Sequencing 8	AGTATGCGGAGTACATCAAG
Sequencing 9	AAGGAGTACCAGGACCTC
Sequencing 10	CCCAGCGAGTATTCAATAAC
Sequencing 11	TTACCGAGCTCAAGGAGC
Sequencing 12	TTTGCTATGCTCACATTGC

Sequencing 13	CAAATGTCAAGCAGCAGG
**Transgenic lines**	
Backbone 1-pBID-UASC-FG-Fwd	CTGCGTCCGCTATCTCTTTC
Backbone 1-pBID-UASC -FG-Rev	gcttttttgtacaaacttgtATACCGGTGCTTGTCATCG
Backbone 2-pBID-UASC-FG-Fwd	ctttcttgtacaaagtgggGACGTAAGCTAGAGGATCTTTG
Backbone 2-pBID-UASC-FG-Rev	GAAAGAGATAGCGGACGC
Tsf1-cDNA-Fwd	tttgtacaaaaaagcaggcttcATGATGTCGCCGCATAAG
Tsf1-cDNA-Rev	tttgtacaagaaagctgggtCACTGCTTGGCAATCTTG
Tsf2-cDNA-Fwd	tttgtacaaaaaagcaggcttcATGGCTAGCAGCCTCGT
Tsf2-cDNA-Rev	tttgtacaagaaagctgggtaGAGCATTGCAACCAGCGAAC
Tsf3-cDNA-Fwd	tttgtacaaaaaagcaggcttcATGCAGTGGCTTACACTTATTT
Tsf3-cDNA-Rev	tttgtacaagaaagctgggtCTAGCCAAACGGCTGAC
Tsf1-sequencing 1-Fwd	ATCACCAAGCTGAAGAACAC
Tsf1-sequencing 2-Fwd	TTCAGTCCACCGATTGTG
Tsf1-sequencing 3-Fwd	AACGACAAAGCCGTTCAG
Tsf2-sequencing 1-Fwd	CTTTCCGACAAACACAATCC
Tsf2-sequencing 2-Fwd	AGATGATCAGTCATTTACCAAG
Tsf2-sequencing 3-Fwd	TTCATGAAGCACACCACG
Tsf3-sequencing 1-Fwd	TTGGTCCCAGTTGCAAGG
Tsf3-sequencing 2-Fwd	TGGTGTTGGTGGATCAGG
Tsf3-sequencing 3-Fwd	TTGTTCCGTGACGACACC
Backbone 1-pBID-UASC-GV-Fwd	CTGCGTCCGCTATCTCTTTC
Backbone 1-pBID-UASC -GV-Rev	CGAGCTCTCCCGGGAATTC
Backbone 2-pBID-UASC-GV-Fwd	TGATATCGCATGCGTGAGCAAG
Backbone 2-pBID-UASC -GV-Rev	GAAAGAGATAGCGGACGCAGC
Evi5^{WT}-cDNA-Fwd	gaattcccgggagagctcgGCCACCATGGCCATGACCCTGACCACAACGAC
Evi5^{WT} -cDNA-Rev	cctcgcccttgctcacgcatgcgatGCTTATCCATTTCCACGGCTC
**Transgenic Evi5^{R160A} line**	
Fragment 1-pBID-UASC-GV-Fwd	CTGCGTCCGCTATCTCTTTC
Fragment 1-Evi5^{R160A}-cDNA-Rev	gcgtgacattgcc**GCC**ACCTATCCCGAGGTGGAG
Fragment 2-Evi5^{R160A}-cDNA-Fwd	acctcgggataggt**GGC**GGCAATGTCACGCCGTATG
Fragment 2-Evi5^{R160A}-cDNA-Rev	cctcgcccttgctcacgcatgcgatGCTTATCCATTTCCACGGCTC
Fragment 3-pBID-UASC-GV-Fwd	TGATATCGCATGCGTGAGCAAG
Fragment 3-pBID-UASC-GV-Fwd	GAAAGAGATAGCGGACGCAGC
**S2 cells transfection**	
pAFW and pAMW Backbone1-Fwd	GGGATCCAGACATGATAAGATAC
pAFW and pAMW Backbone1-Rev	TCAGAGTTGATGCCATTCATG
pAFW and pAMW Backbone2-Fwd	CATGAATGGCATCAACTCTGA
pAFW and pAMW Backbone2-Rev	GAATTCCACCACACTGGAC
Add mCherry to pAFW-Fwd	agtccagtgtggtggaattcttaattaaTTGGTACCGGAgatatcATGGCCATCATCAAGGAGTTC
Add mCherry to pAFW -Rev	gggttagggataggcttaccTTTATACAGCTCGTCCATGCC
Add eGFP to pAFW-Fwd	agtccagtgtggtggaattcttaattaaTTGGTACCGGAgatatcATGGTGAGCAAGGGCGAG
Add eGFP to pAFW-Rev	gggttagggataggcttaccCTTGTACAGCTCGTCCATGCC
Tsf1 cDNA to pAFW with C eGFP-Fwd	agtgtggtggaattcttaatGCCACCATGGACTACAAAGAC
Tsf1 cDNA-for pAFW with C eGFP-Rev	tcgcccttgctcaccatgatCTGCTTGGCAATCTTGTTAGCA
Tsf1 cDNA-no signal-P for pAFW with N 3XFlag and C eGFP-Fwd	ggttattgtctcatgagcggGCCACCATGGACTACAAAG
Tsf1 cDNA-no signal-P for pAFW with N 3XFlag and C eGFP-Rev	cgataaatgggttccatcatGAAGCCTGCTTTTTTGTACAAAC
Tsf2 cDNA- for pAFW with C eGFP-Fwd	agtgtggtggaattcttaatATGGCTAGCAGCCTCGT
Tsf2 cDNA-for pAFW with C eGFP-Rev	tcgcccttgctcaccatgatctaCTTGTCATCGTCATCCTTGTAATCG
Tsf3 cDNA- for pAFW with C eGFP-Fwd	agtgtggtggaattcttaatATGCAGTGGCTTACACTTATTT
Tsf3 cDNA-for pAFW with C eGFP-Rev	tcgcccttgctcaccatgatctaCTTGTCATCGTCATCCTTGTAATC
Evi5 cDNA- for pAFW with C mCherry-Fwd	agtgtggtggaattcttaatATGACCCTGACCACAACG
Evi5 cDNA-for pAFW with C mCherry-Rev	tcgcccttgctcaccatgatctaGCTTATCCATTTCCACGGCT
Evi5 cDNA- for pAFW with C 3XFlag-Fwd	agtccagtgtggtggaattcgccaccatggccATGACCCTGACCACAACG

Evi5 cDNA-for pAFW with C 3XFlag-Rev	gggttagggataggcttaccGCTTATCCATTTCCACGGCT
Rab11 cDNA for pAMW with C 5XMyc Fwd	ccggatcggggtacATGGGTGCAAGAGAAGACGAG
Rab11 cDNA for pAMW with C 5XMyc Rev	cttcaccgctcatgatCTGACAGCACTGTTTGCGC
Fer1HCH cDNA for pAMW with C 5XMyc Fwd	gtgtggtggaattcTCATGAGCGGTGATGCC
Fer1HCH cDNA for pAMW with C 5XMyc Rev	catgtctggatcccCTAGCTCTCCATTTCATTCAAGTCCT
pAFW and pAMW sequencing-1	GCGACACGGAAATGTTGAATAC
pAFW and pAMW sequencing-2	CCTTTTGCTCACATGTTCTTTCC
pAFW and pAMW sequencing-3	ATGGTGAGGTCGCCCAAGCTC
pAFW and pAMW sequencing-4	GTGGTTTGTCCAAACTCATCAATG
Tsf1 cDNA sequencing	AAGAACTCGGACAGCGAC
Tsf2 cDNA sequencing	AATCCAACCGGCCAAGCTG
Tsf3 cDNA sequencing	TTCGGGATCACAAGTCTTCG
Rab11 cDNA sequencing	ACGTTTCACGCGCAATGAATTC
Fer1HCH cDNA sequencing	TGGAGGAGCAGCTCCACGG

[A] primers are listed as the Forward (Fwd) and Reverse (Rev) pairs.

[B] lowercase sequence in primers indicates overlap sequences of the Gibson assembly reaction.
