# Supplementary material for: Drosophila Evi5 is a critical regulator of intracellular iron transport via transferrin and ferritin interactions
Source: Nat Commun. 2024 May 14;15:4045. doi: 10.1038/s41467-024-48165-9 (PMC11094094; doi:10.1038/s41467-024-48165-9)
Supplement: Supplementary file 3 — Description of Supplementary Materials [file 41467_2024_48165_MOESM3_ESM.docx]

**Supplementary Data 1. List of 107 proteins identified in the Evi5 in vivo MALDI-TOF assay and gene ontology analysis.**

Score: The sum of the ion scores of all peptides that were identified.

Coverage: The percentage of the protein sequence covered by identified peptides.

# Proteins: The number of identified proteins in a protein group, that is, the number of proteins displayed in the Protein Group Members view.

# Unique Peptides: The number of peptide sequences that are unique to a protein group.

# Peptides: The total number of distinct peptide sequences identified in the protein group.

# AAs: The number of Amino Acids.

MW [kDa]: Molecular Weight [Kilodalton]

**Supplementary Data 2. List of 75 proteins identified in the Evi5 ex vivo MALDI-TOF assay and gene ontology analysis.**

Score: The sum of the ion scores of all peptides that were identified.

Coverage: The percentage of the protein sequence covered by identified peptides.

# Proteins: The number of identified proteins in a protein group, that is, the number of proteins displayed in the Protein Group Members view.

# Unique Peptides: The number of peptide sequences that are unique to a protein group.

# Peptides: The total number of distinct peptide sequences identified in the protein group.

# AAs: The number of Amino Acids.

MW [kDa]: Molecular Weight [Kilodalton]
