# Supplementary material for: Drosophila Evi5 is a critical regulator of intracellular iron transport via transferrin and ferritin interactions
Source: Nat Commun. 2024 May 14;15:4045. doi: 10.1038/s41467-024-48165-9 (PMC11094094; doi:10.1038/s41467-024-48165-9)
Supplement: Supplementary file 6 — Reporting Summary [file 41467_2024_48165_MOESM6_ESM.pdf]

nature portfolio

Corresponding author(s): Kirst King-Jones

Last updated by author(s): Apr 1, 2024

Reporting Summary

Nature Portfolio wishes to improve the reproducibility of the work that we publish. This form provides structure for consistency and transparency in reporting. For further information on Nature Portfolio policies, see our Editorial Policies and the Editorial Policy Checklist.

Statistics

For all statistical analyses, confirm that the following items are present in the figure legend, table legend, main text, or Methods section.

n/a	Confirmed	
☐	☒	The exact sample size (*n*) for each experimental group/condition, given as a discrete number and unit of measurement
☐	☒	A statement on whether measurements were taken from distinct samples or whether the same sample was measured repeatedly
☐	☒	The statistical test(s) used AND whether they are one- or two-sided<br>*Only common tests should be described solely by name; describe more complex techniques in the Methods section.*
☒	☐	A description of all covariates tested
☒	☐	A description of any assumptions or corrections, such as tests of normality and adjustment for multiple comparisons
☐	☒	A full description of the statistical parameters including central tendency (e.g. means) or other basic estimates (e.g. regression coefficient) AND variation (e.g. standard deviation) or associated estimates of uncertainty (e.g. confidence intervals)
☐	☒	For null hypothesis testing, the test statistic (e.g. *F*, *t*, *r*) with confidence intervals, effect sizes, degrees of freedom and *P* value noted<br>*Give P values as exact values whenever suitable.*
☒	☐	For Bayesian analysis, information on the choice of priors and Markov chain Monte Carlo settings
☒	☐	For hierarchical and complex designs, identification of the appropriate level for tests and full reporting of outcomes
☒	☐	Estimates of effect sizes (e.g. Cohen's *d*, Pearson's *r*), indicating how they were calculated

Our web collection on statistics for biologists contains articles on many of the points above.

Software and code

Policy information about availability of computer code

Data collection	No software was used for data collection.
Data analysis	Microsoft Excel was used throughout for data analysis.

For manuscripts utilizing custom algorithms or software that are central to the research but not yet described in published literature, software must be made available to editors and reviewers. We strongly encourage code deposition in a community repository (e.g. GitHub). See the Nature Portfolio guidelines for submitting code & software for further information.

Data

Policy information about availability of data

All manuscripts must include a data availability statement. This statement should provide the following information, where applicable:

- Accession codes, unique identifiers, or web links for publicly available datasets
- A description of any restrictions on data availability
- For clinical datasets or third party data, please ensure that the statement adheres to our policy

We only have Mass-spec data and accession code for data in Pride database is PXD041626, check "Data availability" for details.

Human research participants

Policy information about studies involving human research participants and Sex and Gender in Research.

Reporting on sex and gender	Not applicable
Population characteristics	Not applicable
Recruitment	Not applicable
Ethics oversight	Not applicable

Note that full information on the approval of the study protocol must also be provided in the manuscript.

Field-specific reporting

Please select the one below that is the best fit for your research. If you are not sure, read the appropriate sections before making your selection.

☒ Life sciences ☐ Behavioural & social sciences ☐ Ecological, evolutionary & environmental sciences

For a reference copy of the document with all sections, see nature.com/documents/nr-reporting-summary-flat.pdf

Life sciences study design

All studies must disclose on these points even when the disclosure is negative.

Sample size	We used 50 animals per replicate for most experiments. Sample size was determined based on previous studies. For experiments where sample numbers are different, the sample size has been described with details in the corresponding method section.
Data exclusions	No data were excluded.
Replication	At least three biological replicates were used for experiments. Where applicable (e.g., qPCR) each biological replicate was tested three times (i.e., three technical replicates for each biological replicate). The mass spec experiments were single replicates, however, because they were from completely different sources (cell culture and animals), we only considered the proteins common to both sets. All attempts at replication were successful.
Randomization	All samples used in this study were used randomly for all experiments.
Blinding	Investigators were blinded to group allocation during data analysis and all sample collections were done blindly and without any bias.

Reporting for specific materials, systems and methods

We require information from authors about some types of materials, experimental systems and methods used in many studies. Here, indicate whether each material, system or method listed is relevant to your study. If you are not sure if a list item applies to your research, read the appropriate section before selecting a response.

Materials & experimental systems

n/a	Involved in the study	
☐	☒	Antibodies
☐	☒	Eukaryotic cell lines
☒	☐	Palaeontology and archaeology
☐	☒	Animals and other organisms
☒	☐	Clinical data
☒	☐	Dual use research of concern

Methods

n/a	Involved in the study	
☒	☐	ChIP-seq
☒	☐	Flow cytometry
☒	☐	MRI-based neuroimaging

Antibodies

Antibodies used	Here is the list of antibodies used in this study: anti-FLAG from Cell signalling (#8146S), anti-Hrs from DSHB (#Hrs27-4), Alexa Fluor 488 from Abcam (#150113), Alexa Fluor 555 from Abcam (#150114), rabbit anti-Myc antibody from Cell Signaling (#2278S), and anti-rabbit HRP from Abcam (#97051).

Validation	All antibodies are commercial, and the provider companies have validated them. Catalogue numbers of all antibodies are mentioned in the relevant method section.

Eukaryotic cell lines

Policy information about cell lines and Sex and Gender in Research

Cell line source(s)	In this study, Drosophila Schneider 2 (S2) cells were used. These cells have been derived from primary cultures of Drosophila embryos.
Authentication	Cells were not authenticated.
Mycoplasma contamination	S2 cells used in this study were negative for Mycoplasma contamination.
Commonly misidentified lines (See ICLAC register)	No misidentified cell lines were used in this study.

Animals and other research organisms

Policy information about studies involving animals; ARRIVE guidelines recommended for reporting animal research, and Sex and Gender in Research

Laboratory animals	In this study Drosophila melanogaster Species (Strain: w1118) was used.
Wild animals	No wild animals were used in the study.
Reporting on sex	Animals sex was selected randomly and none of findings apply to only one sex.
Field-collected samples	No field collected samples were used in the study.
Ethics oversight	This study did not require an ethical approval.

Note that full information on the approval of the study protocol must also be provided in the manuscript.
