# Supplementary figures and images for: Drosophila Evi5 is a critical regulator of intracellular iron transport via transferrin and ferritin interactions
Source: Nat Commun. 2024 May 14;15:4045. doi: 10.1038/s41467-024-48165-9 (PMC11094094; doi:10.1038/s41467-024-48165-9)

corresponding to Figure 6

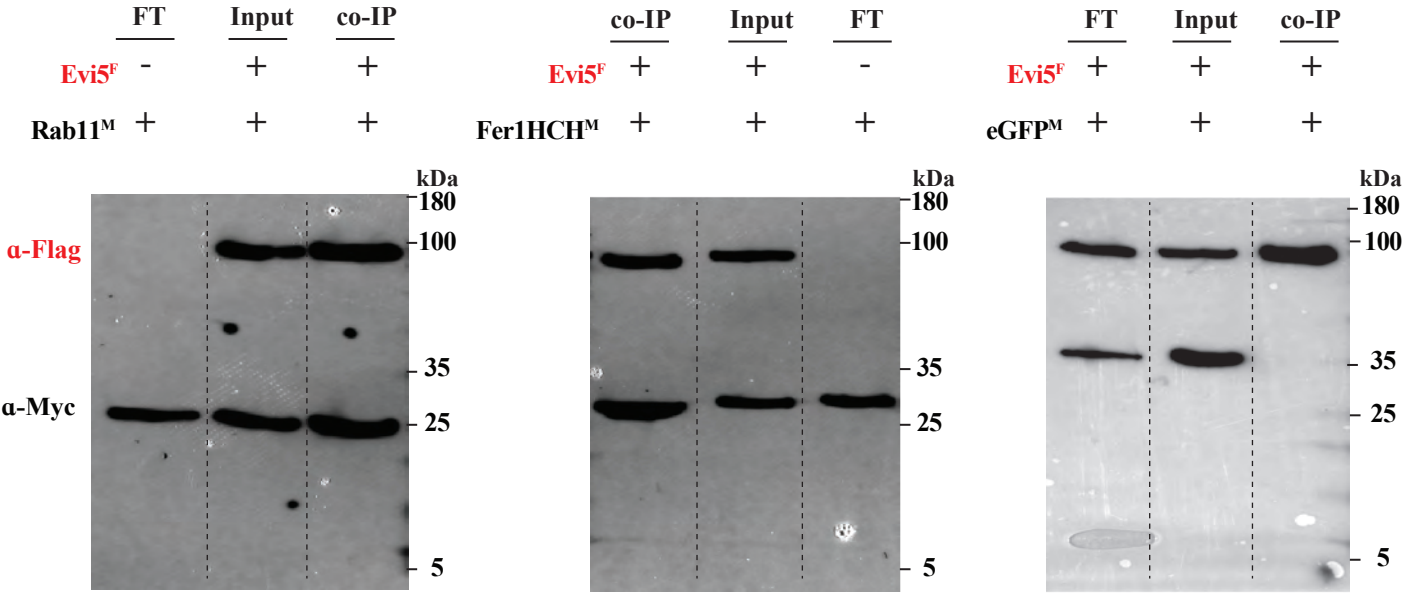

corresponding to Figure S3

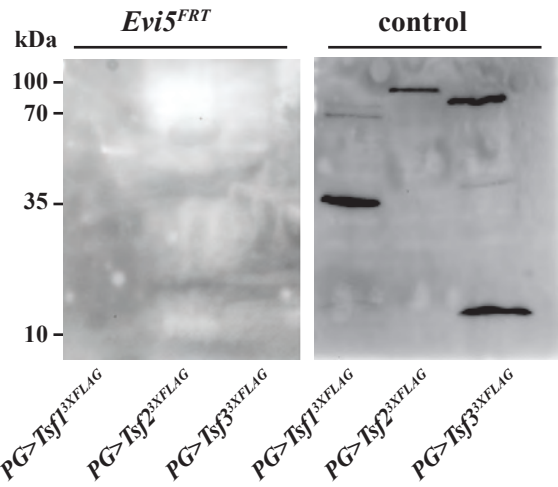

corresponding to Figure S5

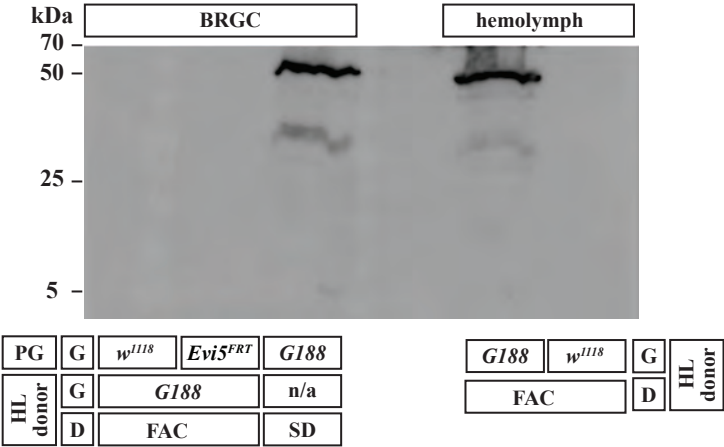

Supplement: Supplementary file 7 — Source Data [file 41467_2024_48165_MOESM7_ESM.zip › Source Data file/Fig. 6B- Fig. S3B- Fig. S5B.pdf]
